# Use of CD200 blockade inhibitor to enhance glioma immunotherapy

**DOI:** 10.1186/2051-1426-3-S2-P38

**Published:** 2015-11-04

**Authors:** Elisabet Mesias, Zhengming Xiong, Elizabeth Pluhar, Michael Olin

**Affiliations:** 1 University of Minnesota, Minneapolis, MN, USA; 2 University of Minnesota, St. Paul, MN, USA

## Background

Utilizing tumors as a source of vaccine antigens in immunotherapy has demonstrated promising results. However, to date, researchers have failed to overcome the complex interplay between the tumor and its surrounding microenvironment resulting in T cell tolerance. The progression to a productive immune response involves a number of immunological checkpoints to be passed. Checkpoints such as CTLA-4 and PD-1 are use to dampen or terminate immune responses, which act as barriers to immunotherapies targeting malignant self-cells that express similar surface antigens as the cells which they derive. To overcome this limitation, the FDA approved three checkpoint inhibitors ipilumimab, pembrolizumab and nivolumab.

## Methods

In our glioma model, we discovered an excess of soluble CD200 protein in the cerebral spinal fluid and cervical lymph nodes, which acts as a checkpoint blockade. CD200 is highly expressed in a variety of human tumors including melanoma and glioblastomas. There are multiple checkpoints blockades including CD200.

## Results

We determinerd that CD200/CD200R interactions upregulate peptidylprolyl isomerase A (PPIA) inhibiting the production of TNF-α and IL-2, induces the formation of myeloid derived suppressor cells (MDSC) and tolergenic antigen presenting cells. We developed a competitive inhibitor of CD200 capable of overcoming CD200 induced suppression. Preliminary data show that these inhibitor peptides inhibit PPIA production and the differentiation of MDSC's, induce lymphocyte infiltration into site of vaccination, upregulate MHC-II and co-stimulatory molecules enhances immunogenicity resulting in a significant survival benefit in our glioma and breast tumor models. Moreover, the use of our inhibitor in our canine spontaneous glioma model resulted in tumor regression and lymphocyte infiltration into the tumor site in our patients. Recently, we developed human candidate peptides for translation to the clinic. Our preliminary data showed that pulsing monocyte derived immature dendritic cells up-regulated MHC-II and CD86 and enhanced IL-12, TNF-b, and chemokine's Rantes, CXCL8, CXCL9 and CXCL10 secretion.

## Conclusions

This study has major implications for the design of translational research approaches and future clinical trials. The proposed study is designed to elucidate and overcome the mechanism of tumor-induced immunosuppression. If our hypothesis is correct, the use of a competitive inhibitor may overcome the suppressive properties of the tumor in both the sentinel lymph nodes as well within the tumor microenvironment ultimately leading to the development of novel therapeutics that increase the efficacy of cancer immunotherapy.

